# Nutritional management during chemotherapy and chemoradiotherapy for advanced esophageal cancer

**DOI:** 10.1007/s10388-025-01117-8

**Published:** 2025-03-29

**Authors:** Yutaka Kimura, Atsushi Gakuhara, Shuichi Fukuda, Yasunari Fukuda, Terukazu Yoshihara, Chikato Koga, Naotsugu Haraguchi, Jin-ichi Hida

**Affiliations:** Department of Surgery, Kindai University Nara Hospital, 1248-1 Otoda-Cho, Ikoma, Nara 630-0293 Japan

**Keywords:** Advanced esophageal cancer, Chemotherapy, Chemoradiation, Nutritional assessment, Nutritional intervention

## Abstract

Advanced esophageal cancer is treated by chemotherapy, radiation therapy, chemoradiotherapy, and immunotherapy. However, the stenosis caused by the tumor and cancer-related chronic inflammation leads to inadequate food intake, weight loss, and nutrition problems. Given that poor pre-treatment nutritional status increases the risks of treatment-related adverse events and a poor prognosis, the nutrition guidelines recommend a pre-treatment nutritional assessment. When malnutrition is present, nutritional interventions, such as dietary guidance and enteral nutrition supplements, provided by the medical team may reduce treatment-related adverse events. However, whether nutritional intervention improves the prognosis is a topic for future research, including randomized controlled trials. This review discusses the literature on nutritional management in patients undergoing chemotherapy and chemoradiotherapy for advanced esophageal cancer.

## Introduction

The 2022 practice guidelines for esophageal cancer edited by the Japan Esophageal Society recommend neoadjuvant chemotherapy (NAC) followed by surgery for advanced esophageal cancer, chemotherapy plus immunotherapy for unresectable esophageal cancer with distant metastasis or invasion of other organs, and chemoradiotherapy (CRT) as an alternative treatment [1]. However, patients with advanced esophageal cancer often develop nutritional disorders and lose weight because of cancer-related chronic inflammation and inadequate food intake resulting from the stenosis caused by the tumor. Given that pre-existing malnutrition can affect adherence with treatment and the prognosis, the European Society for Clinical Nutrition and Metabolism practice guidelines recommend a pre-treatment nutritional assessment and intervention for patients with malnutrition [2, 3]. This paper reviews the literature on the significance of nutritional status and nutritional therapy when administering chemotherapy, CRT, and immunotherapy in patients with advanced esophageal cancer.

## Nutritional assessment before treatment

Patients with advanced cancer typically develop nutritional disturbances and lose weight as a result of both cancer-related metabolic abnormalities and insufficient food intake [4]. Hagi et al. evaluated dietary intake before NAC in patients with advanced esophageal cancer who proceeded to surgery and found that 21.1% had a very poor dietary intake and a dysphagia score of ≥ 3 [5]. Oral intake is even worse in patients with unresectable advanced esophageal cancer, with symptoms of stenosis reported by about half of these patients [6, 7].

The practice guidelines recommend pre-treatment nutritional assessment to screen for cancer-related cachexia, a syndrome that includes poor nutritional status and an enhanced inflammatory response, which may not only be unresponsive to chemotherapy and CRT but also lead to worsening of the patient’s general condition because of adverse events [2, 3].

Several screening tools can be used to assess nutrition status, including the Subjective Global Assessment (SGA), Controlling Nutritional Status (CONUT), which is calculated by scoring serum albumin, the peripheral blood lymphocyte count, and total cholesterol level, the Geriatric Nutrition Risk Index, and the Global Leadership Initiative on Malnutrition criteria. If a patient is found to be malnourished, it is important to provide oral nutritional supplements or intravenous nutrition depending on oral intake status.

## Nutritional indicators (including inflammatory indicators and prognosticators) and prognosis

It has been reported that the more rapid the weight loss and the lower the body mass index before treatment, the poorer the prognosis after treatment is started [8]. It is also known that preoperative sarcopenia is associated with an increased risk of postoperative complications and a poor prognosis. Similarly, in patients undergoing chemotherapy or CRT, sarcopenia is associated with poorer survival (Table 1) [9–13]. Onishi et al. reported a poor prognosis in patients with sarcopenia determined by measurement of skeletal muscle mass by computed tomography in 176 cases of unresectable advanced esophageal cancer (hazard ratio [HR] 1.48, 95% confidence interval [CI] 1.04–2.10) [13].

**Table 1:** Studies of the prognosis in patients with esophageal cancer and sarcopenia

Authors	Year	References	Study design	Number of cases	Treatment	Results
Jogiat UM	2023	9	meta	5 studies	Non-surgical	OS: HR 1.51 (95% CI 1.21–1.89)
Sato S	2018	10	R, CS	48	CRT	Poor 3-year OS (sarcopenia [36.7%] vs non-sarcopenia [63.9%])
Qian J	2022	11	R, CS	213	RT, CRT	OS: HR 1.638 (95% CI 1.113–2.410) PFS: HR 1.509 (95% CI 1.052–2.164)
Mallet R	2020	12	R, CS	97	CRT	OS: HR 2.32 (95% CI 1.25–4.34)
Onishi S	2019	13	R, CS	176	CRT, CT, RT, BSC	OS: HR 1.48 (95% CI 1.04–2.10)

*meta* meta analysis, *R* retrospective, *CS* cohort study, *CRT* chemoradiation therapy, *RT* radiotherapy, *CT* chemotherapy, *BSC* best supportive care, *OS* overall survival, *HR* hazard ratio, *CI* confidence interval, *PFS* progression-free survival

**Fig. 1:**
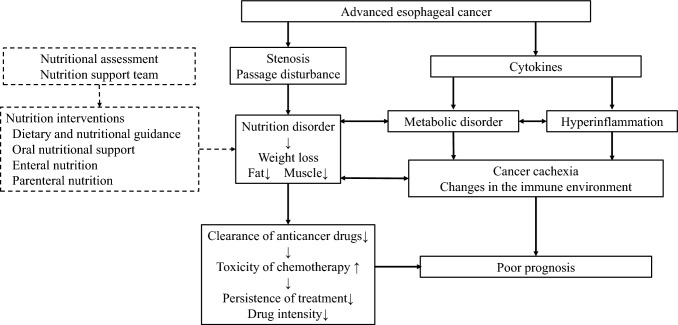
Nutrition and the prognosis in patients with advanced esophageal cancer

A retrospective study of 187 patients with esophageal cancer and recurrent or distant metastases by Zhou et al. showed that the higher the Nutrition Risk Screening score, the poorer the prognosis [14]. Wang et al. similarly found that the prognosis was poor in patients with a high Nutrition Risk Screening score [15]. The Geriatric Nutrition Risk Index, calculated from the serum albumin level and the current/ideal body weight ratio, has been developed as a predictor of complication and mortality rates in the elderly, and the higher the index, the worse the prognosis in patients with advanced esophageal cancer treated by CRT and radiotherapy [16]. As Aoyama et al. have reported, many other reports have been published on the association of nutritional assessment and immune status indices with the prognosis of esophageal cancer (Table 2) [17]. The Glasgow Prognostic Score (GPS), the modified GPS, and the serum C-reactive protein to albumin ratio (CAR) are prognostic markers in patients with esophageal cancer treated by chemotherapy, CRT, and radiotherapy [18–22]. In JCOG0303, a clinical trial of radical CRT for unresectable advanced esophageal cancer by Okuno et al., there was a significant association of the GPS with survival rate (HR 1.22, 95% CI 1.19–3.18) [7]. It has also been found that the higher the neutrophil-to-lymphocyte ratio (NLR), which is a marker of systemic inflammation, the worse the prognosis in patients with esophageal cancer treated by CRT [23–26]. Patients with a high NLR after CRT have also been reported to have a poor prognosis [27]. Moreover, survival was found to be significantly worse in patients with advanced esophageal cancer treated by chemotherapy and CRT if they had a low Prognostic Nutritional Index (PNI; also known as the Onodera Index), which is calculated from the serum albumin level and total lymphocyte count [28–30]. In another study, the prognosis was poorer in patients with esophageal cancer treated by CRT if they have a lower platelet-to-lymphocyte ratio (PLR), a nutritional index that combines inflammatory and immune indices [31]. Therefore, many parameters have been shown to indicate a poor prognosis in the presence of malnutrition in patients with esophageal cancer undergoing chemotherapy and CRT. Cytokines secreted by the tumor affect liver, muscle, and adipose tissue, as well as other sites in the body, leading to poor nutritional status, increased inflammation, and loss of skeletal muscle (Fig. 1). However, the mechanism by which malnutrition and loss of skeletal muscle affect the prognosis requires future research.

**Table 2:** Studies of nutritional indicators (including inflammatory indices) and prognostic factors in patients with esophageal cancer

Authors	Year	References	Study design	Number of cases	Treatment methods	Indicators	Results
Zhou X	2017	14	R, CS	187	CT	NRS	NRS ≥ 3, OS: HR = 1.58 (95% CI: 1.07–2.34)
Wang J	2018	15	R, CS	97	CRT	NRS-2002	NRS ≥ 3, OS: HR = 2.98 (95% CI: 1.39–6.40)
Zhou J	2022	16	meta	3 studies	non-surgical treatment	GNRI	Low GNRI, OS: HR = 2.04 (95% CI: 1.47–2.81)
Crumley AB	2006	18	R, CS	258	CT, BSC	GPS	high GPS, OS: HR = 1.51 (95% CI: 1.22–1.86)
Morikawa T	2014	19	R, CS	111	2nd line CT, BSC	GPS	Low GPS, OS: HR = 0.61 (95% CI: 0.46–0.81)
Ohira M	2015	20	R, CS	91	CRT, Surg	GPS	GPS 1–2, OS: HR = 2.151 (95% CI 1.167–3.966)
Kimura J	2016	21	R, CS	142	CRT	GPS	GPS 2, OS: HR = 2.258 (95% CI 1.494–4.277)
Okuno	2017	7	R, CS	131	CRT	GPS	High GPS, OS: HR 1.22 (95% CI 1.19–3.18)
Zhang H	2019	22	R, CS	266	CRT	CAR	CAR ≥ 0.13, OS: HR 4.344 (95% CI 3.145–5.999)
Yoo EJ	2014	23	R, CS	138	CRT	NLR	NLR ≥ 2, OS: HR 2.115 (95% CI 1.193–3.749), PFS: HR 1.799 (95% CI 1.050–3.083)
Zhou XL	2017	24	R, CS	517	CRT	NLR	NLR ≥ 5, OS: HR 1.856 (95% CI 1.498–2.300), PFS: HR 1.529 (95% CI 1.311–2.025)
Li KJ	2019	25	R, CS	204	CRT	NLR	NLR ≥ 2.64, OS: HR 1.597 (95% CI 1.151–2.215), PFS: HR 1.918 (95% CI 1.406–2.617)
Ho YC	2021	26	R, CS	101	CRT	NLR	NLR ≥ 3.56, OS: HR 2.357 (95% CI 1.115–3.414), PFS: HR 1.918 (95% CI 1.406–2.617)
Matsumoto Y	2018	28	R, CS	191	CT, CRT	PNI	PNI ≥ 43.2, OS: HR 0.93 (95% CI 0.88–0.98)
Dai Y	2019	29	R, CS	106	RT, CRT	PNI	PNI ≥ 48.15, OS: HR 0.537 (95% CI 0.342–0.844)
Xiao L	2021	30	R, CS	193	RT, CRT	PNI	PNI ≥ 47.975, OS: HR 0.584 (95% CI 0.408–0.835)
Tseng RH	2022	31	R, CS	420	CRT	PLR	PLR ≥ 375, OS: HR 1.532 (95% CI 1.143–2.054)

*R* retrospective, *CS* cohort study, meta meta analysis, *P* prospective, *CT* chemotherapy, *CRT* chemoradiotherspy, *BSC* best supportive care, Surg surgery, *RT* radiotherapy, *NRS* nutrition risk score, *GNRI* geriatric nutrition risk index, *GPS* Glasgow prognostic score, *CAR* CRP to albumin ratio, *NLR* neutrophil-to-lymphocyte ratio, *PNI* prognostic nutritional index, *PLR* platelet-to-lymphocyte ratio, *OS* overall survival, *HR* hazard ratio, *CI* confidence interval, *PFS* progression-free survival

Immune checkpoint inhibitors (ICIs) have recently been approved for use in patients with esophageal cancer and there have been several reports on their association with nutritional and immune-related parameters (Table 3). As with conventional chemotherapy, the prognosis has been reported to be better in patients with esophageal cancer treated by ICIs if they have adequate nutritional status, indicated by low CONUT, high GPS, low NLR, high PNI, and low CAR values [32–37]. In a multivariate analysis of ICI-treated cases by Inoue et al., a lower CAR was associated with more adverse events and a higher CAR with a poorer prognosis after treatment with an ICI (HR 10.149, 95% CI 2.664–66.729) [36]. Takegawa et al. also found that the prognosis was poor in 37 patients with previously treated advanced or recurrent esophageal cancer who received nivolumab if they had a PNI < 45 (HR 2.725, 95% CI 1.249–5.947) or a GPS of 1–2 (HR 2.691, 95% CI 1.202–6.022) [37]. It is thought that nutritional status and the systemic inflammatory response may affect the local immune environment, including the tumor, thereby influencing the effect of ICIs. However, the mechanism requires further investigation.

**Table 3:** Studies of the effect of treatment of esophageal cancer with immune checkpoint inhibitors on nutritional indices (including inflammatory indicators) and prognostic factors

Authors	Year	References	Study design	Number of cases	Indicators	Results
Chang L	2022	32	R, CS	69	CONUT, NLR	CONUT score ≤ 1, OS: HR 2.056 (95% CI 1.031–4.098), NLR > 2.24, OS: HR 2.8302 (95% CI 1.235–6.482)
Kim JH	2022	33	R, CS	60	GPS, PNI	GPS 1–2, OS: HR 2.85 (95% CI 1.24–6.56), PNI < 35.93, OS: HR 5.02 (95% CI 1.21–20.76)
Guo JC	2019	34	R, CS	49	NLR	NLR ≥ 6.4, OS: HR 6.31 (95% CI 2.38–16.77), PFS: HR 2.28 (95% CI 1.09–4.74)
Gao Y	2022	35	R, CS	140	NLR	NLR ≥ 5, OS: HR 4.01 (95% CI 2.28–7.06), PFS: HR 1.77 (95% CI 1.12–2.82)
Inoue H	2022	36	R, CS	41	CAR	CAR ≥ 0.119, OS: HR 10.149 (95% CI 2.664–66.729), PFS: HR 2.953 (95% CI 1.344–6.872); CAR < 0.119, AE: OR 9.099 (95% CI 1.997–53.463*)*
Takegawa N	2023	37	R, CS	37	GPS, PNI	GPS 1–2, OS: HR 2.691 (95% CI 1.202–6.022); PNI < 45, OS: HR 2.725 (95% CI 1.249–5.947)

*R* retrospective, *CS* cohort study, *CONUT* controlling nutritional status, *NLR* neutrophil-to-lymphocyte ratio, *GPS* Glasgow prognostic score, *CAR* CRP to albumin ratio, *PNI* prognostic nutritional index, *OS* overall survival, *HR* hazard ratio, *CI* confidence interval, *PFS* progression-free survival, *OR* odds ratio

Given that almost all the studies of various nutritional parameters and the prognosis of esophageal cancer treated by chemotherapy, CRT, and ICI have been retrospective, prospective trials are required in the future.

## Nutritional status and adverse events

Impaired renal function, abnormal liver function, and decreased performance status before treatment are associated with an increased likelihood of serious adverse events after chemotherapy or CRT. Poor renal function and abnormal liver function impair the elimination of anticancer drugs and their metabolites and decreased performance status leads to decreased metabolism, which in turn leads to serious adverse events. Loss of skeletal muscle mass has been associated with a high incidence of chemotherapy-induced adverse events in many types of cancer [38, 39]. Considering that skeletal muscle is an important organ involved in the metabolism of anticancer drugs such as 5-fluorouracil (5-FU), it is thought that a decrease in skeletal muscle mass increases the risk of adverse events because of decreased clearance of these agents [40].

Malnutrition has often been reported to be associated with the development of adverse events and decreased compliance with chemotherapy and CRT for esophageal cancer (Table 4). A study by Hagi et al. in which 434 patients with esophageal cancer received docetaxel + cisplatin + 5-FU (DCF) as an initial treatment. found that those with inadequate pre-treatment food intake and a high dysphagia score of 3–4 had significantly worse nutritional status and a significantly higher incidence of grade ≥ 3 febrile neutropenia and diarrhea [5]. Multivariate analysis in a study by Ishida et al. also identified that loss of skeletal muscle was a risk factor for grade ≥ 3 adverse events (odds ratio [OR] 9.53, 95% CI 1.09–83.1) in 165 patients with esophageal cancer who underwent NAC [41]. There have also been reports of sarcopenic obesity, or sarcopenia with obesity, being associated with greater risk of serious adverse events [42–45].

**Table 4:** Studies of the relationship between nutritional status and adverse events in patients receiving treatment for esophageal cancer

Authors	Year	References	Study design	Number of cases	Treatment methods	Indicators	Results
Hagi T	2019	5	R, CS	434	CT	Dysphagia score	Dysphagia score 3–4, FN: OR 6.24 (95% CI 3.15–12.36), grade 3–4 diarhea: OR 2.92 (95% CI 1.63–5.22)
Ishida T	2019	41	R, CS	165	NAC	Sarcopenia	Low PMI, grade 3–4 AEs, OR 9.53 (95% CI 1.09–83.1)
Tan BH	2015	42	R, CS	89	NAC	Sarcopenia	Low SMI, DLT: OR 2.954 (95% CI 1.230–7.094)
Panje CM	2019	43	P, CS	61	NAC, NACRT	Sarcopenia	Low SMI, grade 3–4 AEs = 83.3% vs high SMI, grade 3–4 AEs = 52.4% (p = 0.041)
Anandavadivelan P	2016	44	R, CS	72	NAC	Sarcopenic obesity	Low SMI and BMI ≥ 25, DLT: OR 5.54 (95% CI 1.12–27.44)
Dijksterhuis WPM	2019	45	R, CS	88	CT	Sarcopenic obesity	Low SMI, PN grade ≥ 2: OR 3.82 (95% CI 1.20–12.18)
Matsumoto Y	2018	28	R, CS	191	CT, CRT	PNI	Hematologic toxicity, grade 1–2: PNI = 47.5 ± 5.8 vs grade 3–4: PNI = 40.3 ± 6.7 (*p* < 0.001)
Hsueh WH	2022	46	R, CS	123	NACRT	NLR, Alb	NLR ≥ 3.1 and/or Alb < 4.1: increased oral mucositis and infections with grade 3 or higher

*R* retrospective, *CS* cohort study, *meta* meta analysis, *P* prospective, *CT* chemotherapy, *NAC* neoadjuvant chemotherapy, *NACRT* neoadjuvant chemoradiotherapy, *CRT* chemoradiotherapy, *PNI* prognostic nutritional index, *NLR* neutrophil-to-lymphocyte ratio, *Alb* serum albumin level, *FN* febrile neutropenia, *OR* odds ratio, *CI* confidence interval, *PMI* psoas muscle index, *AE* adverse event, *SMI* skeletal muscle index, *DLT* dose-limiting toxicity, *BMI* body mass index, *PN* peripheral neuropathy

Severe (grade ≥ 3) adverse events, especially hematological toxicity, are more common in patients with a low PNI [28, 30]. Hsueh et al. reported that patients undergoing CRT for esophageal cancer had a significantly higher incidence of grade ≥ 3 serious adverse events and a lower treatment completion rate if they had a high NLR and a low serum albumin level [46]. Therefore, pre-treatment malnutrition increases the risk of adverse events with chemotherapy and CRT and causes treatment interruptions, so there is a need for pre-treatment nutritional assessment and consideration of nutritional intervention if necessary [2, 3]. Furthermore, food intake is reduced during chemotherapy and CRT because of gastrointestinal toxicity and other factors, making it likely that the patient's nutritional status will deteriorate even further. In view of reports that loss of skeletal muscle increases the toxicity of treatment, nutritional intervention is also needed during treatment to prevent loss of weight and skeletal muscle [47].

## Effectiveness of nutritional interventions

Nutritional interventions include counseling and nutritional guidance by dietitians, oral nutritional support, enteral nutrition, and parenteral nutrition. The effects of these nutritional interventions have been examined in numerous clinical studies, many of which have used nutritional status and treatment toxicity as endpoints (Table 5). Some studies found that survival was longer after CRT for esophageal cancer in patients with poor nutritional status (indicated by a Nutrition Risk Index score of < 100) who received either dietary nutritional guidance, oral nutritional supplements, or enteral nutrition than in their counterparts who received no nutritional intervention. However, few studies have used survival as an endpoint [48].

**Table 5:** Studies of the effects of nutritional intervention in patients on treatment for esophageal cancer

Authors	Year	References	Study design	Number of cases	Treatment methods	Nutritional intervention	Results
Cox S	2016	48	R, CS	31	CRT	DA, ONS, TF (NRI < 100)	DA: HR 0.12 (95% CI 0.03–0.51), ONS: HR 0.13 (95% CI 0.04–0.39), TF: HR 0.13 (95% CI 0.03–0.50)
Qiu Y	2020	50	RCT	96	CRT	NST vs none	Nutritional status and quality of life maintained, fewer cases of radiation esophagitis and skin disorders, and a shorter hospital stay
Wang SA	2023	51	RCT	36	CRT	NST vs none	Nutritional status and quality of life maintained, lymphocyte counts preserved, and a shorter hospital stay
Lu Z	2021	52	RCT	328	CT	NST (2 weeks before treatment) vs none	OS: HR 0.68 (95% CI 0.510–0.90), PFS: HR 0.80 (95% CI 0.62–1.04), quality of life maintained
Miyata H*、*Kita R	2012	53*、*54	RCT	91	NAC	EN vs PN	Grade 3–4 neutropenia: OR 0.28 (95% CI 0.11–0.69), Declining SMI: OR 0.09 (95% CI 0.03–0.25)
Furuta M	2019	55	R, CS	51	CRT	EN vs TPN	Decrease in grade 3–4 neutropenia and FN, albumin is maintained
Wang SA	2018	56	R, CS	104	CT, CRT	TF vs ONS	Nutritional status maintained, reduction of esophagitis, less bone marrow suppression
Xu YJ	2015	57	R, CS	59	NACRT	DA plus walking	Muscle strength and nutritional status maintained
Halliday LJ	2023	58	R, CS	51	NACRT	DA plus exercise	SMI maintained
Christodoulidis G	2023	59	R, CS	92	NACRT	Exercise	CT completion rate: OR 10.93 (95% CI 1.044–114.460)
Miyata H	2017	60	RCT	61	NAC	Omega-3FA EN vs omega-3FA poor EN	Less oral mucositis, less abnormal liver function
Vasson MP	2014	61	RCT	37	CRT	IMN vs EN	Nutritional status and functional capacity maintained (Karnovsky index, WHO/ECOG score*)*
Tanaka Y	2021	62	RCT	113	NAC	ED (1 week before treatment) vs none	Grade ≥ 2 oral mucositis: HR 0.4 (95% CI 0.2–0.9), nutritional status maintained
Kanda C	2021	63	RCT	71	NAC	ED (after starting treatment) vs none	Transferrin maintained. No difference in AEs
Tanaka Y	2022	64	meta	5 studies	CT, CRT	ED	Oral mucositis: OR 0.35 (95% CI 0.12–0.99)
Ishikawa T	2016	65	RCT	33	CT, CRT	ED vs azulene oral rinse	Lean body weight maintained, no difference in oral mucositis
Hiura Y	2012	66	RCT	40	NAC	Ghrelin vs placebo	Dietary intake and appetite maintained, reduction in loss of appetite and nausea
Motoori M	2022	67	RCT	81	NAC	Synbiotics plus EN vs prophylactic antibiotics	reduction in grade 3–4 neutropenia, favorable RDI, good compliance for CT

*R* retrospective, *CS* cohort study, *RCT* randomized-controlled trial, *meta* meta analysis, *CRT* chemoradiotherapy, *CT* chemotherapy, *NAC* neoadjuvant chemotherapy, *NACRT* neoadjuvant chemoradiotherapy, *DA* dietary and nutritional advice, *ONS* oral nutritional support, *TF* tube feeding, *NRI* nutrition risk index, *NST* nutrition support team, *EN* enteral nutrition, *PN* parenteral nutrition, *TPN* total parenteral nutrition, *FA* fatty acid, *IMN* immunomodulatory nutrition, *ED* elemental diet, *HR* hazard ratio, *CI* confidence interval, *QOL* quality of life, *OS* overall survival, *PFS* progression-free survival, *OR* odds ratio, *SMI* skeletal muscle index, *FN* febrile neutropenia, *Alb* serum albumin level, *AE* adverse event, *RDI* relative dose intensity

It is difficult to improve nutritional status and survival in patients with cancer, including those with esophageal cancer, by providing a single nutritional intervention before chemotherapy, and it is recommended that multidisciplinary interventions should be provided by a nutrition support team [2, 3, 49]. A small randomized controlled trial (RCT) showed that for patients with esophageal cancer undergoing CRT, regular monitoring of dietary intake and provision of dietary guidance and nutritional recommendations by a nutrition support team that included a dietitian was effective for not only maintaining energy intake and nutritional status but also reducing the risk of adverse events [50, 51]. Furthermore, an RCT in 328 patients with esophageal or gastric cancer in China showed that multidisciplinary pre-treatment intervention, including from dieticians and psychologists, improved nutritional status and reduced anxiety and was associated with better overall survival (HR 0.68, 95% CI 0.58–0.90) [52].

The recommendation is to use enteral rather than intravenous nutrition if possible [2, 3]. Miyata et al. reported an RCT in which they compared enteral versus intravenous nutrition in patients undergoing NAC for esophageal cancer [53, 54]. They found that enteral nutrition was more effective and that although there was no difference in daily energy intake between the two groups, the enteral nutrition group had a significantly lower rate of loss of skeletal muscle during treatment and a reduced incidence of hematological toxicity. Enteral nutrition has been reported to be more effective than total parenteral nutrition in maintaining nutritional status and reducing hematological toxicity even in patients undergoing radical CRT for esophageal cancer [55]. In view of reports suggesting that enteral nutrition via a nasal feeding tube maintains nutritional status during CRT and decreases hematological toxicity to a greater extent than orally administered nutrition, forced feeding via a nasal feeding tube should be considered in patients with inadequate food intake [56]. Moreover, some reports suggest that nutritional intervention with addition of exercise is more effective than nutritional intervention alone in terms of maintaining nutritional status and skeletal muscle and completion of chemotherapy [57–59].

Immune-modulating preparations containing n-3 fatty acids, such as eicosapentaenoic acid, arginine, glutamine, and nucleic acids, which are thought to activate the immune system, and elemental supplements containing L-glutamine and essential amino acids can also be used as nutritional supplements. One RCT found significantly fewer cases of oral mucositis and abnormal liver function in patients who received n-3 fatty acid-rich nutritional supplements than in those who received regular nutritional supplements during NAC [60]. Another RCT that compared immune-modulating nutritional supplements containing arginine, docosahexaenoic acid, eicosapentaenoic acid, and nucleic acids with conventional nutritional supplements during CRT for esophageal cancer reported that functional capacity (Karnofsky performance status, World Health Organization/Eastern Cooperative Oncology Group score) and nutritional status were better in patients who received immune-modulating nutritional supplements [61]. An RCT by Tanaka et al. investigated the effect of elemental nutritional supplements on adverse events in patients with esophageal cancer treated by DCF. Patients who started taking elemental nutritional supplements (80 g, 600 kcal) 1 week before initiation of chemotherapy were significantly less likely to develop oral mucositis (OR 0.382, 95% CI 0.168–0.870) [62] and experienced less weight loss, hematological toxicity, and elevation of C-reactive protein. However, there have been reports of elemental nutritional supplements not effectively suppressing adverse events when initiated after the start of chemotherapy [63]. A meta-analysis revealed that elemental nutritional supplements significantly suppressed oral mucositis in patients undergoing treatment for cancer (OR 0.25, 95% CI 0.10–0.61) [64]. In another study, 80 g of an elemental nutritional supplement not only reduced oral mucositis but also maintained lean body mass during chemotherapy and CRT [65].

Ghrelin is a hormone secreted mainly by the stomach that stimulates secretion of growth hormone and increases appetite. An RCT that investigated the effect of ghrelin in patients receiving chemotherapy for esophageal cancer that included cisplatin found that ghrelin resulted in more food intake, prevented a decline in prealbumin and transferrin levels, and was associated with significantly fewer adverse events involving anorexia and nausea [66]. Anamorelin hydrochloride, which has ghrelin-like effects in patients with cancer cachexia, is now available but its effects during chemotherapy await further study.

In recent years, it has become clear that the intestinal microbiota plays an important role in the treatment of cancer. Motoori et al. reported the results of an RCT that compared the adverse event rate in patients with esophageal cancer undergoing preoperative DCF according to whether they received prophylactic antibiotics or synbiotics plus enteral nutrition [67]. They found that administration of synbiotics plus enteral nutrition maintained the intestinal environment and prevented grade 4 neutropenia and grade ≥ 2 diarrhea. Further research on the role and efficacy of synbiotics in esophageal cancer patients treated by ICIs as well as chemotherapy is awaited.

## Conclusions

Patients treated with chemotherapy or CRT for advanced esophageal cancer require nutritional assessment before starting treatment because their nutritional status is compromised by symptoms, in particular stenosis, and poor nutritional status is associated with a worse prognosis. Nutritional interventions before and during treatment have been reported to help maintain nutritional status and reduce adverse events during the course of treatment, but whether these interventions lead to an improved prognosis requires further research.

## Data Availability

Data is available upon reasonable request.
